# Uniform and Non-uniform Perturbations in Brain-Machine Interface Task Elicit Similar Neural Strategies

**DOI:** 10.3389/fnsys.2016.00070

**Published:** 2016-08-23

**Authors:** Michelle Armenta Salas, Stephen I. Helms Tillery

**Affiliations:** SensoriMotor Research Group, School of Biological and Health Systems Engineering, Arizona State UniversityTempe, AZ, USA

**Keywords:** learning, adaptation, neuroprosthetics, neural control, neural dynamics

## Abstract

The neural mechanisms that take place during learning and adaptation can be directly probed with brain-machine interfaces (BMIs). We developed a BMI controlled paradigm that enabled us to enforce learning by introducing perturbations which changed the relationship between neural activity and the BMI's output. We introduced a uniform perturbation to the system, through a visuomotor rotation (VMR), and a non-uniform perturbation, through a decorrelation task. The controller in the VMR was essentially unchanged, but produced an output rotated at 30° from the neurally specified output. The controller in the decorrelation trials decoupled the activity of neurons that were highly correlated in the BMI task by selectively forcing the preferred directions of these cell pairs to be orthogonal. We report that movement errors were larger in the decorrelation task, and subjects needed more trials to restore performance back to baseline. During learning, we measured decreasing trends in preferred direction changes and cross-correlation coefficients regardless of task type. Conversely, final adaptations in neural tunings were dependent on the type controller used (VMR or decorrelation). These results hint to the similar process the neural population might engage while adapting to new tasks, and how, through a global process, the neural system can arrive to individual solutions.

## 1. Introduction

Motor and skill learning are closely related terms often used to describe the acquisition and retention of behaviors through repeated practice (Shmuelof and Krakauer, 2011). Brain-machine interfaces (BMIs) have proven to be a unique environment in which to study the neural correlates of this motor learning. Early reports of BMIs focused heavily on changes in neural tuning as subjects adapted to these peculiar output systems and learned to control the movement of an effector that they had never before experienced (Wessberg et al., 2000; Serruya et al., 2002; Taylor et al., 2002; Carmena et al., 2003): clearly these systems would require learning and adaptation to even operate. This challenge to neuroprosthetics has become an opportunity in which BMIs provide a novel environment to directly probe and measure the brain's plasticity. These BMI systems can be used to create a wide variety of learning challenges, from altering the decoders output to directly changing the contribution each neural signal will have in this output, where the scope of the perturbations will not to be limited by the physics of actual movement. BMIs also simplify the task of simultaneously studying different brain structures during learning and adaptation. For example, BMIs have been used to test the ability of subjects to learn new tasks (Shadmehr and Mussa-Ivaldi, 1994; Taylor et al., 2002; Carmena et al., 2003; Hochberg et al., 2006; Velliste et al., 2008; Orsborn et al., 2012) or to adapt to new environments (Tong and Flanagan, 2003; Tanaka et al., 2009). It has been possible, using BMIs, to identify changes in the properties of individual neurons after learning a task (Taylor et al., 2002; Carmena et al., 2003; Lebedev et al., 2005; Zacksenhouse et al., 2007; Ganguly and Carmena, 2009; Ganguly et al., 2011; Chase et al., 2012), as well as the existence of constraints within the neural circuitry which can hamper skill learning (Paz and Vaadia, 2004; Jarosiewicz et al., 2008; Ranganathan et al., 2014; Sadtler et al., 2014). In other words, these BMI systems give vast opportunities to uniquely challenge neural circuitry and probe the neural basis of learning and adaptation (Ganguly and Carmena, 2009; Ranganathan et al., 2014; Sadtler et al., 2014).

Given the present evidence of plasticity in BMI systems, it is clear that a given neural ensemble is able to adapt to different decoders, suggesting these systems are able to generate strategies that solve a number of diverse challenges. These decoders need not follow exact representations of muscle activation or movement (Ganguly and Carmena, 2009), although the speed of adaptation seems to relate to how closely the decoder follows this relationship (Ganguly and Carmena, 2009; Ganguly et al., 2011; Sadtler et al., 2014). These different adaptations have system-wide impact, i.e., changes in neural properties are measured throughout the entire neural ensemble (Jarosiewicz et al., 2008; Ganguly and Carmena, 2009; Ganguly et al., 2011; Chase et al., 2012; Wander et al., 2013; Addou et al., 2014; Okun et al., 2015). For example, in tasks with visuomotor rotations or force-field perturbations, the system appears to solve the problem, and respond to the perturbations mostly in a uniform manner across the neural population (Tong and Flanagan, 2003; Rokni et al., 2007; Tanaka et al., 2009). However, different groups have shown that not all neurons change in the same manner: the amount of adaptation may correlate with the properties required by the new controller (Paz and Vaadia, 2004; Jarosiewicz et al., 2008; Chase et al., 2012).

It has been shown that subjects can adapt to uniform perturbations to their movements (Krakauer et al., 2000; Tong and Flanagan, 2003), most likely driven by uniformly shifting the neuronal ensemble tuning properties (Rokni et al., 2007; Tanaka et al., 2009). Neural systems can also adapt to certain non-uniform perturbations by finding a workable solution based on a relatively uniform response across the ensemble, although the overall ensemble dynamics can change in unpredictable ways (Jarosiewicz et al., 2008; Ganguly and Carmena, 2009). It remains unclear whether the differences in the measured adaptations are due to the nature of the challenges (e.g., visual rotations, force fields, shuffled decoders, etc.), or due to the overall difficulty of the task. For example, it is not clear at this point how the system would respond to a perturbation that only impacted a focal set of cells which did not have an equivalent visuomotor rotation. How does the learning process compare across these different tasks: does the neural system try to apply the same adaptation strategies, or does it engage different adaptation processes? Do these strategies change the properties of the signals or can they also alter the underlying dynamics of the area of cortex undergoing adaptation? Lastly, while there is evidence that a neural ensemble can alternate BMI control between a normal and a shuffled decoder (Ganguly and Carmena, 2009), it has not been shown that a given set of neurons can process two very different kind of perturbations to the decoder, which could have great influence in understanding the limitations of motor learning, and might impact the design of motor rehabilitation paradigms. In this study, we will address the questions of whether the adaptation to uniform and non-uniform perturbations have similar effects on neural tuning when these perturbations are directly applied in BMI paradigms, and whether these adaptations change the underlying input signals which condition the activity of the measured neural units.

Previous studies have tracked changes in the tuning of neural systems in BMI tasks (Rokni et al., 2007; Jarosiewicz et al., 2008; Ganguly et al., 2011). These results range from substantial variations in the preferred directions of neural units, where neurons display systematic shifts in their tuning (Rokni et al., 2007), or local shifts correlated to an introduced perturbation (Jarosiewicz et al., 2008), to emergence of a stable tuning across the neural ensemble (Ganguly and Carmena, 2009). Building from these studies, we hypothesize that to solve distinct motor learning tasks, the neural population will respond globally rather than locally, varying the behavior of the entire neuronal ensemble, even when the perturbation is only across a limited subset of that ensemble. Otherwise, the first task for the ensemble will be to solve the credit assignment problem (i.e., identify which cells have changed properties in the decoder): this strikes us as an enormously difficult task given the sparse sampling of the neural systems which characterizes BMIs (e.g., recording a hundred channels of the tens of millions of neurons participating in the control of movement). Instead, we propose that motor neural circuitry will exhibit changes across the entire neuronal ensemble when challenged with different uniform and non-uniform perturbations, and then using trial and error, determine a workable solution to the current perturbation, not unlike the error-noise-learning trade discussed by Rokni et al. (2007). Importantly, we expect that different tasks will elicit similar adaptation strategies throughout the learning process, even when the final neural solutions are not similar between the tasks.

Here we report the response of the motor cortical system to two different control perturbations: one global and uniform, and a second that is focal and non-uniform. In contrast with previous studies (Paz and Vaadia, 2004; Jarosiewicz et al., 2008; Ganguly and Carmena, 2009), the subjects were trained in brain control only, and did not perform any overt movements for either task. Similar to Ganguly and Carmena (2009) we used the same decoder across several days. We recorded and characterized the neural behavior during and after learning, anticipating that any tuning changes induced by these tasks would be reflected in the final adaptations displayed by the entire neuronal ensemble.

## 2. Methods

### 2.1. Experimental set-up and recordings

All experimental protocols were in accordance with the Guide for the Care and Use of Laboratory Animals, and approved by the Arizona State University Institutional Animal Care and Use Committee. We implanted two non-human primates (*Macaca mulatta*) with six bilateral (monkey O) and four unilateral (monkey M) 16-channel micro-wire arrays (Tucker Davis Technologies, Inc.), in the hand and arm regions of the motor and dorsal premotor cortices. The coordinates of the cortical regions of interest were acquired with previously described methods (McAndrew et al., 2012), and verified by visual inspection of the implantation sites during surgery. A 96-channel recording system (Plexon, Inc., Dallas TX) was used to capture, filter, and sort single and multi-unit activity. Units were sorted using voltage threshold and waveform shape detection. Action potentials that met both the threshold and waveform criteria were registered as spikes, and sorted as a neural unit. Data from all channels were captured at 40 kHz and saved for *post-hoc* analysis.

**Figure 1:**
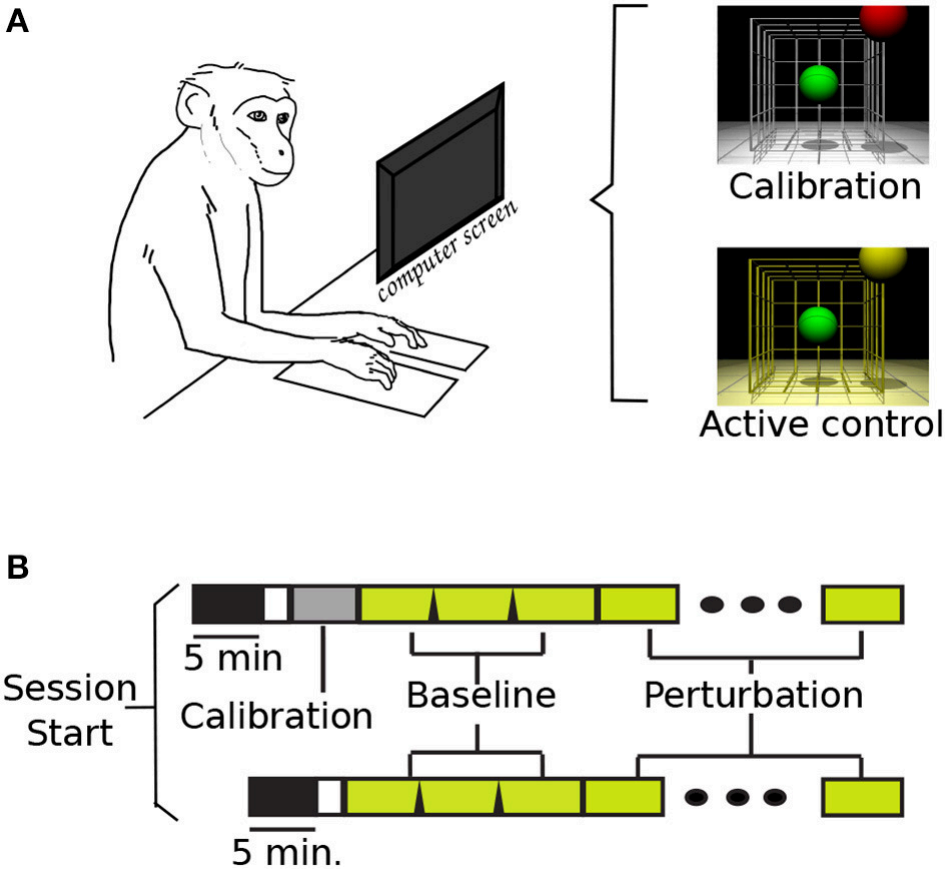
**Task set-up and time line**. **(A)** NHP in primate chair set-up, and VR screen with display lighting during calibration and active brain control. **(B)** Task time-lines (with and w/o calibration block): task-off, calibration, baseline, and perturbation.

The monkeys were trained to sit on a primate chair and to observe three dimensional center-out movements of a computer cursor in a 3D monitor (SeeReal Technologies), while keeping their hands on pads located on the desk immediately in front of the primate chair (Figure 1A). The task required continuous contact with both left and right hold-pads in order to operate. At least once weekly, and more frequently when recording conditions were changing, the monkeys performed calibration trial blocks. In these calibration blocks, the animals observed the cursor moving automatically to each of the eight targets at a constant speed. In each trial, the cursor took approximately 9.5 s to move through a straight path to the target. Neural activity during these epochs exhibited adequate directional tuning to initialize a population vector. From this point, the motion of the cursor was controlled by neural activity using a modified version of the population vector algorithm (PVA) from Georgopoulos et al. (1986). Changes in recording conditions were noted if a neuron's waveform was no longer recorded in a channel, or if the sorted neurons no longer met the previously established sorting criteria. Supplementary Table 1 shows a summary of the decoder updates for each subject, and the total amount of sessions with new and fixed decoders. Overall, the subjects learned a total of twelve (monkey O) and five (monkey M) different calibration maps during the experiments described here.

The tasks were organized by blocks of 32 trials. During those blocks, the control code remained fixed: cursor and target diameters, successful/failed trials criteria and cues, and inter-trial times. The cursor and targets were differently colored spheres: the cursor was green for all conditions, and the target was red during calibration blocks and yellow during all brain control blocks. Different background lighting in the virtual-reality (VR) display was also used during the task: white for calibration, and yellow for baseline and perturbations (Figure 1A). Two cases were considered a failed trial: if the subjects did not reach the target in a set time (13–22 s), or if the cursor went out of bounds. These bounds were experimenter selected, and they were the virtual limits where the cursor would no longer be visible in the task display. Similarly, the time-out values were updated during initial task training, and left fixed once the perturbations were introduced. Between trials, the cursor and target were blanked for 0.8–2.4 s inter-trial interval (ITI). The ITI was empirically varied to allow subjects to receive a juice reward after successful trials.

Figure 1B displays the two possible timelines in a session. The upper timeline illustrates the event sequence with a calibration session; while the bottom timeline represents the days when the monkeys started directly with active brain control. Supplementary Table 2 summarizes the total number of recording sessions for each subject, the total of sessions where a new calibration map was used, and those where the previous map was kept. The first 5 min of recording were captured without any task display: the monkeys sat quietly on the primate chair with the monitor off. These data were used in a separate study comparing background activity with task-based activity. Each session consisted of three baseline blocks, 32 trials each, followed by blocks of perturbations (average perturbed blocks = 4.7, s.d. = 1.1). Once the perturbation was introduced, it was kept constant throughout the remainder of the session. If the monkey removed either hand from the copper plates, the task would pause until both plates were pressed, and the task would resume with the cursor back at the center position.

### 2.2. Neural decoding for brain control

We adapted the PVA to decode the movement of the cursor from neural activity. Our choice of the population vector was largely on the ease with which changes in neural tuning can be quantified. There are many other possible algorithms, and smoother and better control has been reported with other linear and non-linear decoders such as Kalman filter, particle filters or Bayesian approaches (Carmena et al., 2003; Wu et al., 2003; Hochberg et al., 2006; Velliste et al., 2008; Hochberg et al., 2012; Orsborn et al., 2012); however any parametric changes in neural tuning using these other decoders are more difficult to decipher. Furthermore, in a direct comparison of several of these decoders, most subjects can compensate online for poor preferred directions distributions while using PVA, and have similar performance across different decoders (Koyama et al., 2010).

To begin, we assumed the neurons had cosine tuning profiles as stated in Equation (1).

(1) $$\begin{array}{r} f_{i}=b_{0}+m_{i}cos\theta , \end{array}$$

where *f*_*i*_ refers to the ith neuron's firing rate, *b*_0_ to the tonic activity of the ith neuron, *m*_*i*_ is the depth of modulation for the ith neuron, and θ is the angle between the *preferred direction* (PD) of the cell and the intended movement direction. Since these neurons also display a preferential tuning when observing movement in a VR environment (Wahnoun et al., 2006), we calculated the preferred directions from recordings during the calibration trials. We estimated each neuron's *calibration preferred direction* (cPD) using a multivariate linear regression, which related the neuron's change in firing rate from their baseline to the target direction while the cursor automatically moved during the calibration trials. We excluded the data when the task was paused (i.e., subjects lifted hands from hold pads) and during the ITI. The firing rate was calculated online from the spiking activity of non-overlapping 50 ms bins and a running mean for each neuron using a rectangular kernel (Nawrot et al., 1999). The estimated directions were converted to unitary vectors and used to control the movement of the cursor, unless the 90% confidence interval for all three coefficients spanned zero. During active brain control, the subjects O an M had on average 32.19 (s.d. 7.48) and 20.17 (s.d. 2.48) units tuned to the task, respectively. These cPDs were used to compute the population vector shown in Equation (2).

(2) $$\begin{array}{r} {\vec{PV}}_{t}=\overset{N}{\underset{i=1}{\sum }}\left(f_{i}-\bar{f_{i}}\right)\vec{cPD_{i}}, \end{array}$$

where $\vec{cPD_{i}}$ is the ith neuron's preferred direction, *f*_*i*_ is the instantaneous firing rate, $\bar{f_{i}}$ is the baseline firing rate, computed as a running mean for each cell across the entire block, and $\vec{PV_{t}}$ is the final population vector for that time step. The population vector was calculated every 50 ms, and the position of the cursor was updated using this vector. The population vector was smoothed using a two time-step window moving average filter. Equation (3) displays the formula used to update the cursor position.

(3) $$\begin{array}{r} \vec{C_{t}}=\vec{C_{t-1}}+\left[\vec{PV_{t}}\cdot g\cdot \left(1-h\right)+\vec{Tar_{t-1}}\cdot h\right]s\cdot L, \end{array}$$

where vectors $\vec{C_{t}}$ and $\vec{C_{t-1}}$ refer to the current and previous cursor position, respectively. The vector $\vec{PV_{t}}$ represents the current smoothed population vector, and the scalar *g* is the population vector gain factor (μ_*O*_ = 34.94, s.d. 18.6; μ_*M*_ = 412.58, s.d. 4.36). Additional parameters include active assistance factor (*h*_*O*_ = 8% and *h*_*M*_ = 0.8%), speed gain (*s*: experimenter selected), and length of the population vector (*L*). The vector $\vec{Tar_{t-1}}$ is the direction to target from the previous cursor position, normalized to be a unitary vector.

For the second monkey we set parameter *L* to unity, instead of using the length of the PV. Since this term was scaling down the cursor movements. However, with either of the controllers the subjects had good control of the cursor movement, and the decoded PV dictated the overall speed of the movements. The experimenter selected values were adjusted such that the cursor would not easily shoot out of the virtual workspace range.

### 2.3. Uniform and non-uniform perturbations to task

We introduced two types of perturbation to the task: (1) a visuomotor rotation (VMR), around the axis into the monitor, of 30° in CCW and CW directions; and (2) a decorrelation perturbation (DeCorr), in which we chose a subset of the pairs of neurons with highest peak cross-correlation values, and constrained their contribution to the population vector to be uncorrelated by assigning them orthogonal preferred directions.

**Figure 2:**
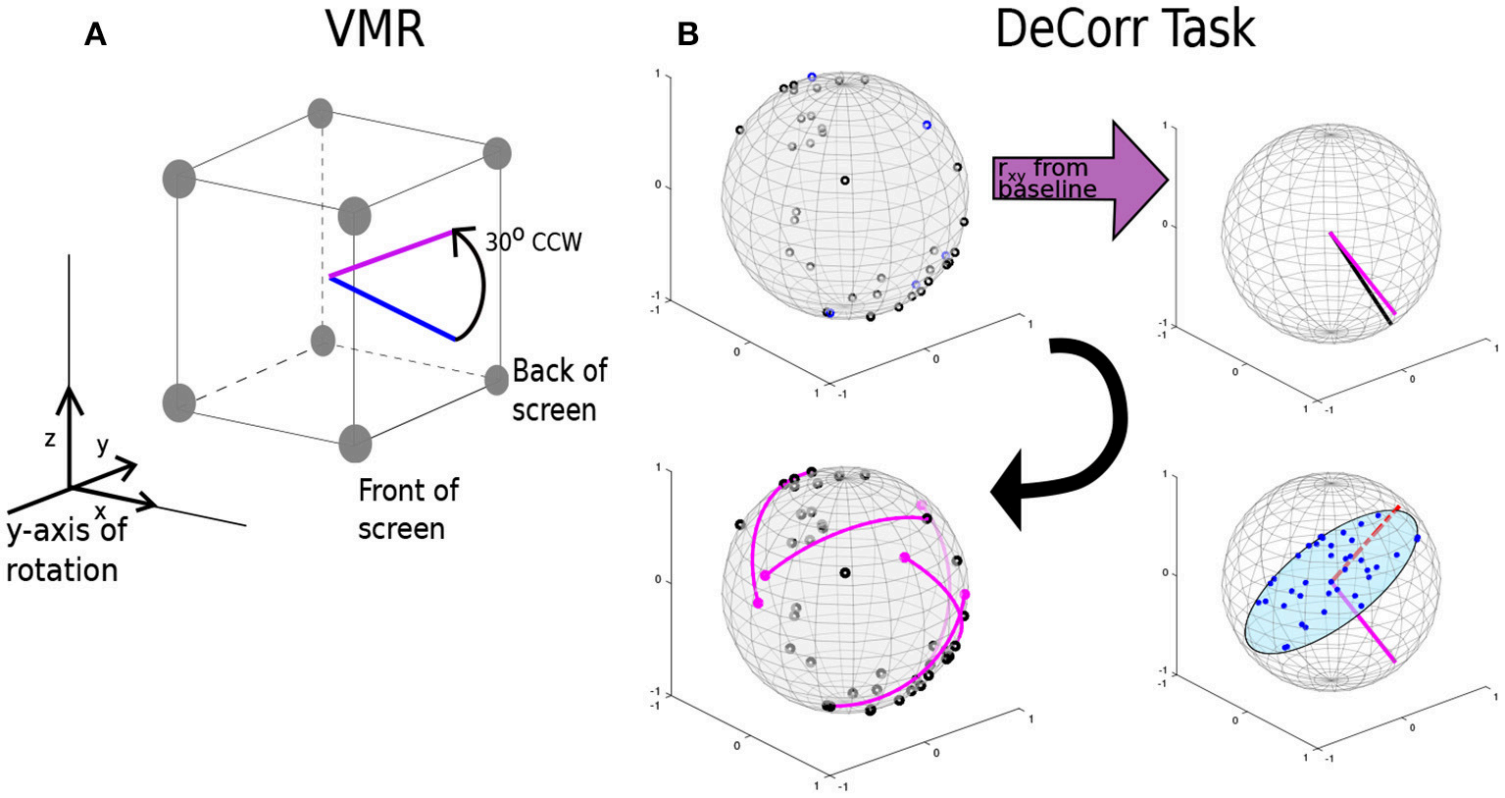
**VMR and DeCorr Perturbations**. **(A)** Diagram displays the VMR applied to the original population vector (blue) and the resulting rotated vector (magenta), which was used to update the cursor movement. **(B)** DeCorr procedure (read clockwise from top left corner): from a given cPD distribution the cross-correlation coefficients were computed (*r*_*xy*_), and top correlated pairs were then selected. A neuron was randomly selected from the pair (magenta vector), and the rest of the cPDs were projected into an orthogonal plane. The cPD for the selected neuron was rotated toward the region of the plane with fewer neurons (red dashed vector). This process was repeated for a small number (4–6) of neuron pairs.

In the VMR task, we selected the antero-posterior axis as our reference vector, with positive directions into the VR display. We then rotated the computed PV using a rotation matrix derived from the Rodrigues' rotation formula Equation (4).

(4) $$\begin{array}{r} R=\left[\begin{array}{ccc} cos\theta & 0 & sin\theta \\ 0 & 1 & 0\\ -sin\theta & 0 & cos\theta \end{array}\right], \end{array}$$

where θ is the angle of rotation around the selected axis. The rotation was applied to the PV before updating the cursor position. A positive theta yielded a CCW rotation, and a negative theta a CW rotation. Figure 2A displays the rotation of an arbitrary movement vector in CCW direction, where the blue vector is the initial intended movement, and the magenta vector is the outputted movement after the rotation. After a new decoder calibration, we randomly selected the direction of the perturbation (CW o CWW), and used this until the next decoder update. We selected the *y*-axis as reference for the rotation, which places the rotation in the plane of the display screen.

For the DeCorr perturbation, we intended to disrupt the internal dynamics of the neural population and introduce a difficult challenge to the control paradigm. We used the cross-correlation function between the firing rates of neurons to identify functional connections between the cells (Vaadia et al., 1995; Salinas and Sejnowski, 2001). We considered that selecting these highly correlated pairs allowed us to directly interfere with the ensemble dynamics, without relying on the individual contribution each neural unit has in the decoder.

Figure 2B shows the DeCorr perturbation procedure: we calculated the cross-correlations between all neuron pairs from activity recorded in the baseline intervals. We then selected the pairs which displayed the highest maximum cross-correlations. One neuron was randomly selected from each pair, and the cPD of the selected neuron was rotated to a new direction orthogonal to the starting cPD. To select the specific direction within that orthogonal plane, we projected the cPDs of the entire ensemble to that same plane, and computed the circular histogram count of the projected neurons, using five degree bins. We then identified the bin of the plane with the fewest projected cPDs. The new cPD for the selected cell was chosen to fill the most substantial gap in that orthogonal plane. This process was repeated four to six times, with no single cell involved in more than a single rotation. Supplementary Figure 1A shows the distribution of angles between all the cPDs for a given decoding map of monkey O and monkey M before (black) and after (purple) the perturbation. Supplementary Figure 1B displays the distribution of angle differences between the rotated pairs before (black) and after (purple) the DeCorr perturbation of the same ensemble cPDs for both monkeys. The sum of the individual rotations did not result in substantial rotation to the output (two-sample test, circular data, *p* > 0.05, Zar, 1996).

### 2.4. Control for chance performance

In order to test whether the subjects were able to rely on active assistance to perform the tasks, we simulated the task offline using the stored firing activity during baseline, VMR and DeCorr trials, but manipulated the relationships between firing and device motion in two ways. First, we used the actual cPDs and randomly assigned them to actual neural units. Our goal was to keep the same directional distribution and neuron firing rates, but to alter the relationship between neurons and their preferred directions. Second, we again shuffled cPD and neural units relationship, but used the mean firing activity from brain control trials toward the same target. The purpose was to check for significant target hits if the subjects had not modulated his firing activity. In both cases, we recalculated the population vector using Equation (2), and updated the cursor position using Equation (3). All task parameters were kept the same as those used during active brain control. From these simulations, we computed the estimated target hits and angular errors between population vector and target direction. These controls provided a measure of the extent to which the cursor motion was due to active assistance, and how much was due to subjects modulating their activity. In cases using modulated firing rates, we would observe only 2 or 3 target hits in 96 trials. If only the help was included (no firing rate modulation), there were no target hits (see Supplementary Figure 2 for details).

### 2.5. Changes in tuning properties and neural ensemble dynamics

We expected tuning to change between tasks, and specifically, we expected preferred direction to change (Taylor et al., 2002; Jarosiewicz et al., 2008; Chase et al., 2010). Therefore, a key measure of changes in the neural system was observed by calculating the preferred directions associated with action in the virtual task (*action PDs* or aPDs), using the same tuning equation shown in Equation (1). Many of our main results reflect differences in the aPDs between conditions. As a primary measure of changes in cell properties, we calculated the angle between the aPDs of baseline and perturbed trials for both successful and failed trials.

To measure any possible changes in neuronal ensemble dynamics, we calculated the cross-correlation function during both the baseline trials and the perturbations using 1 ms bins and a Gaussian kernel of 200 ms (Nawrot et al., 1999). We compared the peak cross-correlation coefficients for each trial across the different paradigms. We used one-way analysis of variance to test the effect that task type (baseline and perturbation) had in these peak cross-correlation coefficients, in both the rotated and non-rotated neuron pairs. We also compared the peak cross-correlation shifts from baseline during the first phases of task learning to those from when the monkeys were fully adapted to the tasks.

The tasks are three-dimensional, and so the control of the tasks is over-specified by the neural ensemble. Because of this, it is possible that each of the tasks is eventually controlled in separate neural spaces. To capture this, we used a dimensionality reduction algorithm to identify control manifolds specific to each task, and to explore whether the neural activity could be expressed in terms of latent dimensions (Rubin and Thayer, 1982; Sadtler et al., 2014). This method uses the expectation-maximization algorithm (Rubin and Thayer, 1982) to iteratively estimate a subspace or manifold that related the activity of each of the recorded neurons to the solution space for a given phase of the task (see Supplementary Material). This space may indicate latent variables driving the activity of the recorded neurons (Santhanam et al., 2008; Yu et al., 2009) or describe further functional connections between the neural units. We used existing algorithms and MATLAB scripts (Yu et al., 2009; Cowley et al., 2013) to estimate these intrinsic manifolds and latent variables from our raw neural data. We estimated these manifolds using a fixed number of latent dimensions (*n* = 12) across the different trials. The number of dimensions was selected from cross-validation with initial data calculated with the DataHigh toolbox. The software (Cowley et al., 2013) iterates across possible latent dimension values and computes the log-likelihood from the binned firing activity (50 ms bins). We used the the average number of dimensions at which the log-likelihood function was maximized (see Supplementary Figure 8).

Finally, we computed the principal angles (PAs) between these estimated manifolds for baseline and perturbation trials. These PAs capture the intersections between subspaces. For dimensions that two subspaces share, the PAs are near zero. For dimensions that the two manifolds do not share, the PAs are closer to 90°, indicating that the subspaces are distinct. We used an algorithm and MATLAB function which allows for precise estimation of small angles between subspaces (Knyazev and Argentati, 2002). We measured the changes in the distribution of the PAs as subjects improved performance in both perturbations, and performed one-way analysis of variance to test whether task type (baseline, VMR, and DeCorr) and performance (number of correct trials) had an effect on these PAs.

## 3. Results

We trained both subjects on both perturbations. For monkey O, we were able to observe full adaptation back to initial levels of performance in both tasks. For monkey M, we were only able to complete adaptation in the VMR task. After 3 days of training with the DeCorr task, the recording implants failed (loss of neurophysiological signals). After the subjects had been trained with both types of perturbation, we measured the variations in behavior and performance across days where perturbations were constant. For all the performance measurements of VMR sessions, the rotation directions (CCW and CW) were merged, we did not find significant differences in performance or movement errors among them (one-way ANOVA, *p* > 0.8). Pertinent corrections for rotation direction were made when measuring the angle shift in aPDs, and movement errors.

**Figure 3:**
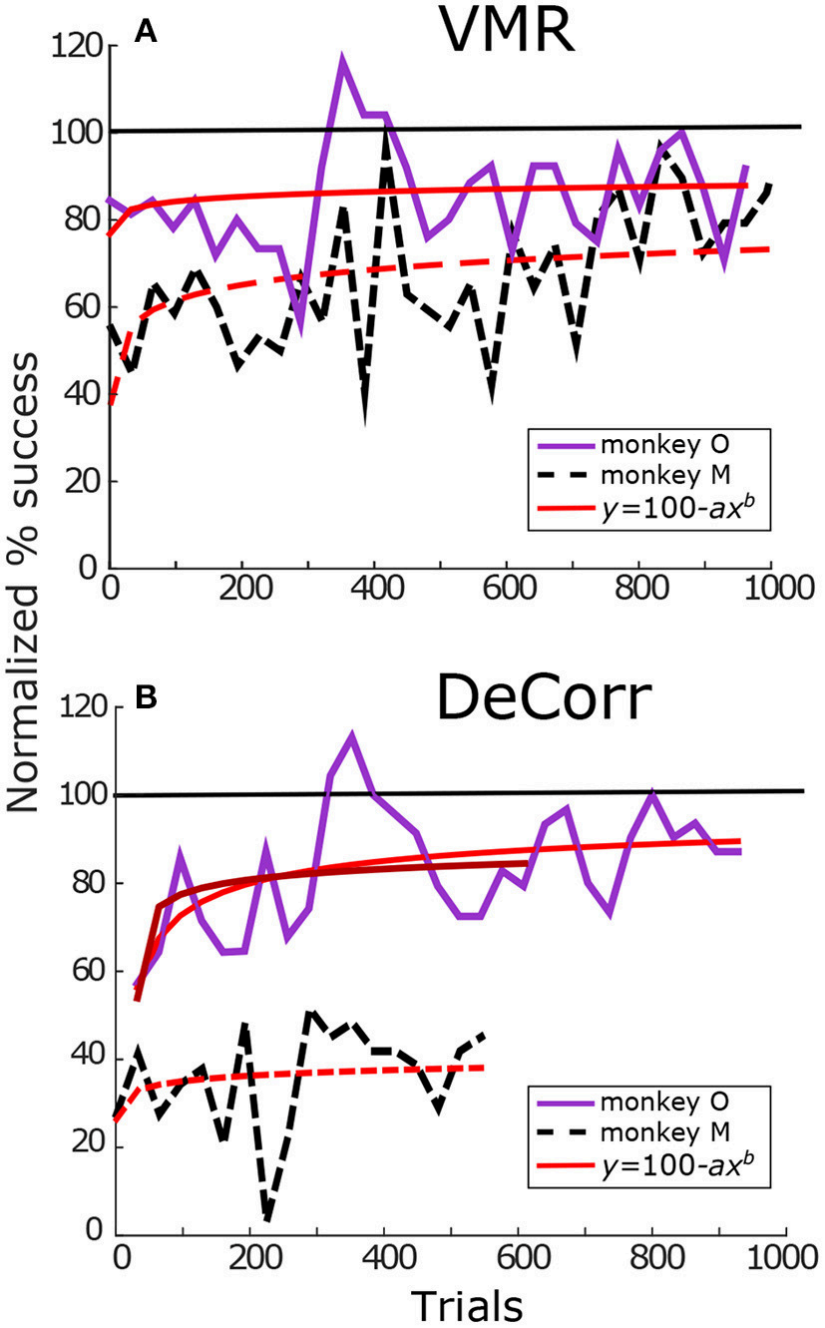
**Tasks performance**. **(A)** VMR success for monkey O (solid purple) and monkey M (dashed black). Trials span across different days. Percentage success was normalized with maximum performance in baseline trials. The success rate was calculated per block (32 trials/block). Red trace shows fit for Wright's learning curve (*y* = 100−*ax*^*b*^). with coefficients *a*_*O*_ = 22.11, *b*_*O*_ = −0.08 and *a*_*M*_ = 58.1, *b*_*M*_ = −0.10. **(B)** DeCorr performance for monkey O and monkey M in solid purple and dashed black lines, respectively. Trials span across different days with the same perturbed cPDs. Coefficients *a*_*O*, 1_ = 47.43, *b*_*O*, 1_ = −0.19, *a*_*O*, 2_ = 46.09, *b*_*O*, 2_ = −0.17, and *a*_*M*_ = 74.91, *b*_*M*_ = −0.03. The first fit of monkey O used the complete trial set, while the second one used partial trial set similar in length to that of monkey M. Format is the same as top panels. (monkey M had less data in DeCorr task, trials span for ~500 trials).

Figure 3 summarizes task success of the perturbations for both monkeys. Figure 3A displays the percentage success for VMR sessions for monkey O (solid purple lines) and M (dashed black lines). Illustrated trials belong to sessions where the cPDs distributions and the perturbation were kept constant. Percentage success was normalized according to the maximum success rate during the baseline trials. The red trace represents model fitting of a modified Wright's (1936) learning curve (*y* = 100−*ax*^*b*^), the coefficient *b* represents how quickly or slowly the subjects improved in the task. Values close to zero mean the learning was slow, while values close to negative one mean the subjects adapted quicker as they performed more trials. We observed similar learning rates for both subjects in VMR trials, as shown in Figure 3A. Subject M had a slightly faster adaptation with an estimated coefficient *b* = −0.10, while subject O had an estimated *b* = −0.08. Similarly, Figure 3B displays the increase in performance in the DeCorr trials for both subjects. Subjects O and M had estimated coefficients of *b* = −0.19 and *b* = −0.03, respectively.

As we can observe in Figure 3B, monkey M performed fewer trials of the DeCorr task, although he showed improvement in the task, he did not reach accuracy similar to the baseline trials and displayed slower learning than monkey O. However, the improvements were similar along the initial learning phases, and we will compare the initial phases of learning between subjects. For example, during the first couple of sessions (~500 trials) both had an increase of approximately 20% of initial performance (Figure 3B), and there was not a significant difference between the normalized performance of these trials (one-way ANOVA, *F* = 0.7, *p* > 0.4). In order to make comparisons of this initial learning, we separately fit monkey O's initial data (first ~ 500 trials) to the modified learning curve. This separate fit is shown in the darker red solid line in Figure 3B, with an estimated coefficient of *b* = −0.17.

### 3.1. Tuning properties variations with learning

As a first measurement of responses in the neural signals, we compared the firing rates across days where we used the same decoding cPDs maps for cursor control, and we did not find significant differences between baseline and the perturbations (one-way ANOVA, *p* > 0.1). We also looked for specific differences between the firing activity of the rotated and non-rotated neurons in the DeCorr task, and we also found no significant variations between these groups. As a next step, to show that the changes measured would not be due to random variations in the aPDs but due to task adaptation, we analyzed the shifts in aPDs during baseline trials, and tracked them across days were the decoders were fixed. Supplementary Figure 4 shows an example of the trends we measured across trials for both subjects. Overall, the aPDs estimated during baseline trials stabilized over time (*p* > 0.1, circular data one-way ANOVA), returning to directions similar to those measured during previous sessions.

**Figure 4:**
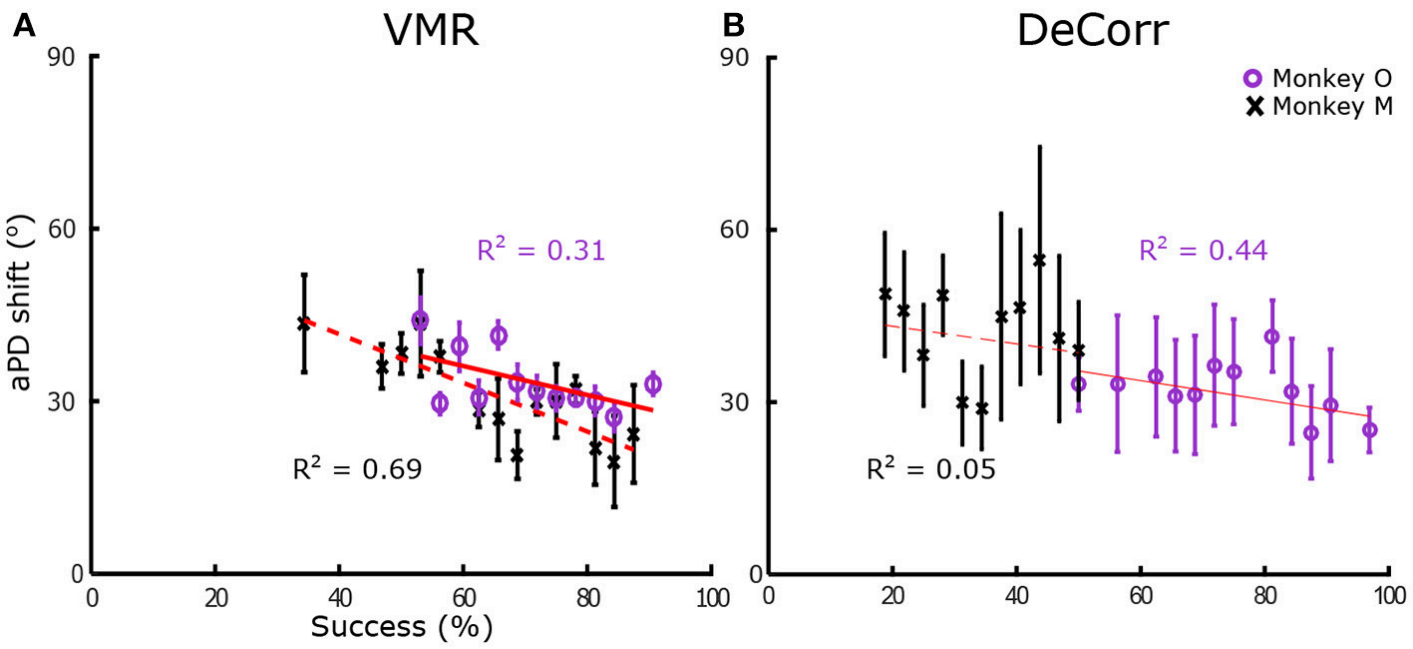
**Preferred direction changes during learning**. **(A)** Average arc length vs. percentage success in VMR task for monkey O (purple circles) and monkey M (black cross), error bars display standard error. Red traces display fit for linear model (*y* = *ax* + *b*), with coefficients *a*_*O*_ = −0.229, *b*_*O*_ = 51.222, *a*_*M*_ = −0.401, *b*_*M*_ = 58.614. **(B)** Average length vs. percentage success in DeCorr task for both subjects, error bars display standard error. Red traces display fit for linear model, with coefficients *a*_*O*_ = −0.172, *b*_*O*_ = 43.831, *a*_*M*_ = −0.172, *b*_*M*_ = 46.295.

In order to quantify tuning changes in individual signals while the subjects adapted to the task, we calculated the angles between aPDs of baseline and perturbed trials. We measured these aPDs at two different stages, when the subjects were adapting to the tasks, and when they had steady improvements in performances. To measure changes during learning, we used sets of 16 trials, measuring the angle shift between subsequent trial sets until subjects had reached between 90 and 100% accuracy within those trials. Figure 4 shows the average shift between aPDs for VMR and DeCorr trials for monkey O (purple) and M (black). We fitted simple linear first order models to the average aPD shifts, shown in the red traces. We observed a decreasing trend for both task types, but did not observe any significant differences in the aPD variations of rotated and non-rotated neurons during the initial stages of learning (performance <65%) for either subject (one-factor ANOVA, circular data. Zar, 1996). For monkey M, the DeCorr trials did not have a strong fit to any model but did display a slight decreasing trend (see Figure 4B).

**Figure 5:**
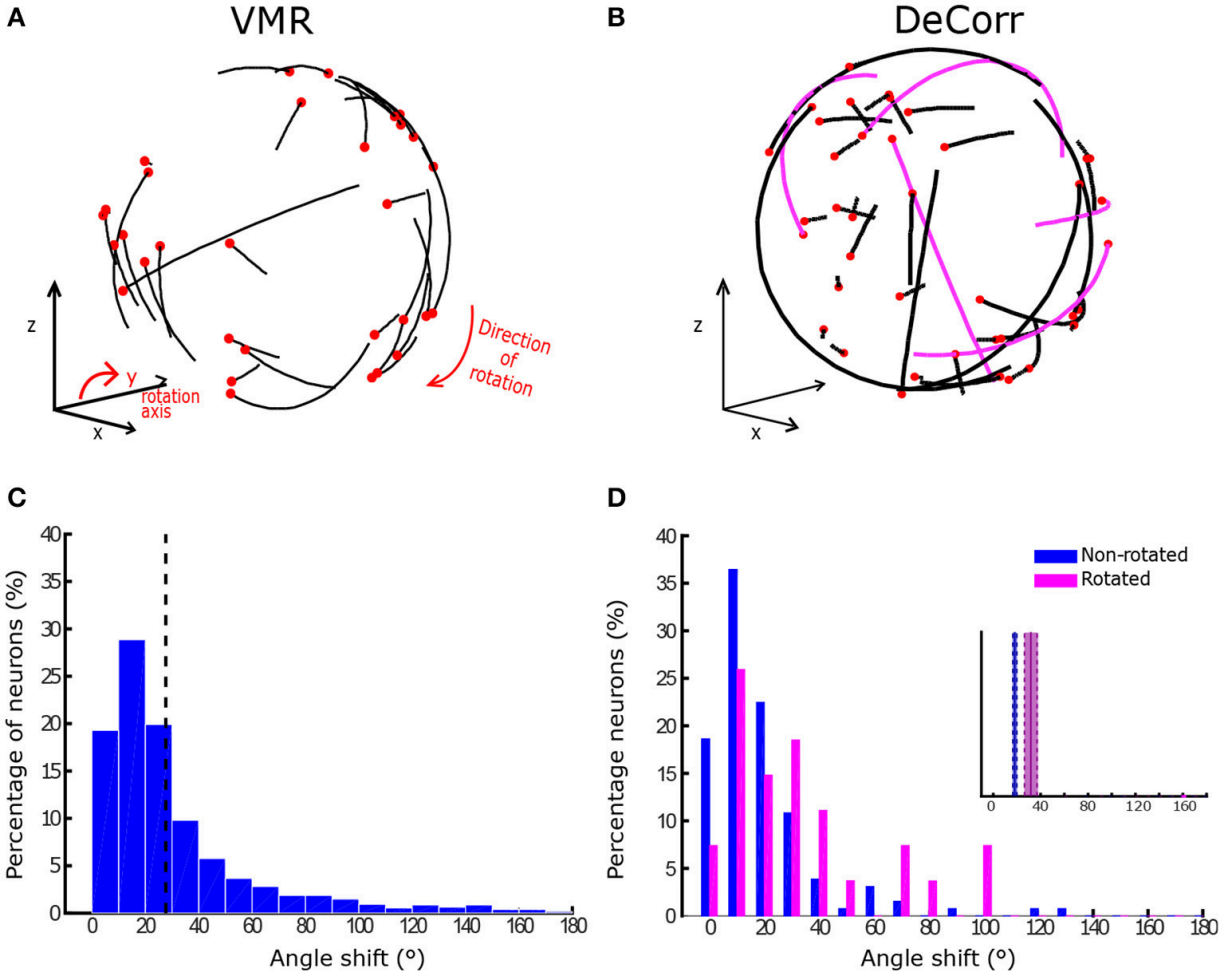
**Preferred direction changes in VMR and DeCorr task**. **(A)** aPDs shifts during VMR task (CW direction): aPDs during baseline (black end) and after perturbation (red dot) are displayed for each neuron used for brain control. **(B)** aPDs changes during DeCorr task (5 rotated pairs): aPDs during baseline (black end) and after perturbation (red dot) are displayed for each neuron used in brain control, magenta trajectories highlight the rotated neurons. **(C)** Shift in neurons aPDs, pooled data are displayed for all VMR sessions for monkey O. Dashed gray line illustrates average shift. **(D)** Shift in neurons aPDs, pooled data for all DeCorr trials for monkey O. Blue bars show data for non-rotated neurons, and magenta bars display data for rotated neurons.

In order to estimate the final aPDs once the monkeys had learned the perturbations, we used only successful trials across different days (200+ trials) where a fixed decoder was used. Figure 5A displays examples of aPD shifts during VMR trials for monkey O in CW direction, where the black end of each trajectory represents the baseline aPD, and the red end shows the aPD during perturbation. Similarly, Figure 5B shows the change in aPDs in the DeCorr trials, the magenta trajectories highlight the rotated neurons during the perturbation. Figure 5C displays changes in VMR aPDs across all days for monkey O. In the majority of the sessions, the aPDs displayed an average angle shift of 30° in the direction of the induced rotation (*p* < 0.05, one-sample test for mean angle; Zar, 1996). On the other hand, Figure 5D shows the distribution of aPD shifts for the rotated (magenta) and non-rotated (blue) cells for the same subject in the DeCorr task. We observed a significant difference between these sub-populations (*p* < 0.01, two-sample test for mean angle; Zar), with a larger shift in the rotated neurons. For the VMR task, we observed similar behaviors in monkey M's aPDs, but not during the DeCorr task (see Supplementary Figure 5). Monkey M had an overall 30° rotation in the VMR trials, but not a significant difference between the rotated and non-rotated sub-populations in the DeCorr task, although the non-rotated neurons had on average larger shifts.

### 3.2. Changes in population dynamics during learning

A key goal in this experiment was to measure whether the tasks, and specially the DeCorr task, resulted in changes in the internal dynamics of the ensemble. Our first measure of changes was to determine whether the overall profile of the cross-correlations between neurons of interest changed with the task. Thus, we compared changes in the peak cross-correlations from the neurons used to control the movement of the cursor while the subjects adapted to the task, using all the trials during these sessions. Here we found that during the majority of the sessions there was a significant change in peak cross-correlations between baseline and DeCorr trials (19/24 sessions, see Supplementary Table 2). For half of the DeCorr sessions, there was a significant drop in cross-correlation coefficients after the perturbation was introduced for both subjects (12/24 sessions, one-way ANOVA, *p* < 0.01). During these sessions, we also observed an increase in the coefficients of all cell pairs as subjects improved performance (see Supplementary Figure 7); however this trend was not present across all days. We also compared changes in correlation between the rotated and non-rotated neurons, and did not find any significant differences between them during most sessions for both subjects (16/24 sessions, *p* ≥ 0.1). These results suggest that the network dynamics are changed during learning, but these changes are not unique to the rotated neurons.

We thought also to measure the state of the ensemble by examining the spaces in which the ensemble encoded movement. To this end we applied factor analysis methods to discern key dimensions of the neural control (Yu et al., 2009; Sadtler et al., 2014). We estimated manifolds which captured the observed firing patterns among the neural ensembles with a smaller set of latent dimensions. We found that with at most 12 dimensions we could capture the key elements of neural activity that were associated with control of the cursor. When we examined the manifolds corresponding to baseline performance and those associated with the perturbations, we found that there was a great degree of overlap between the manifolds, reflected in small principal angles (PAs) between axes of the manifolds. There were generally only one or two dimensions which were distinct between manifolds in the perturbed cases and those from the baseline cases.

**Figure 6:**
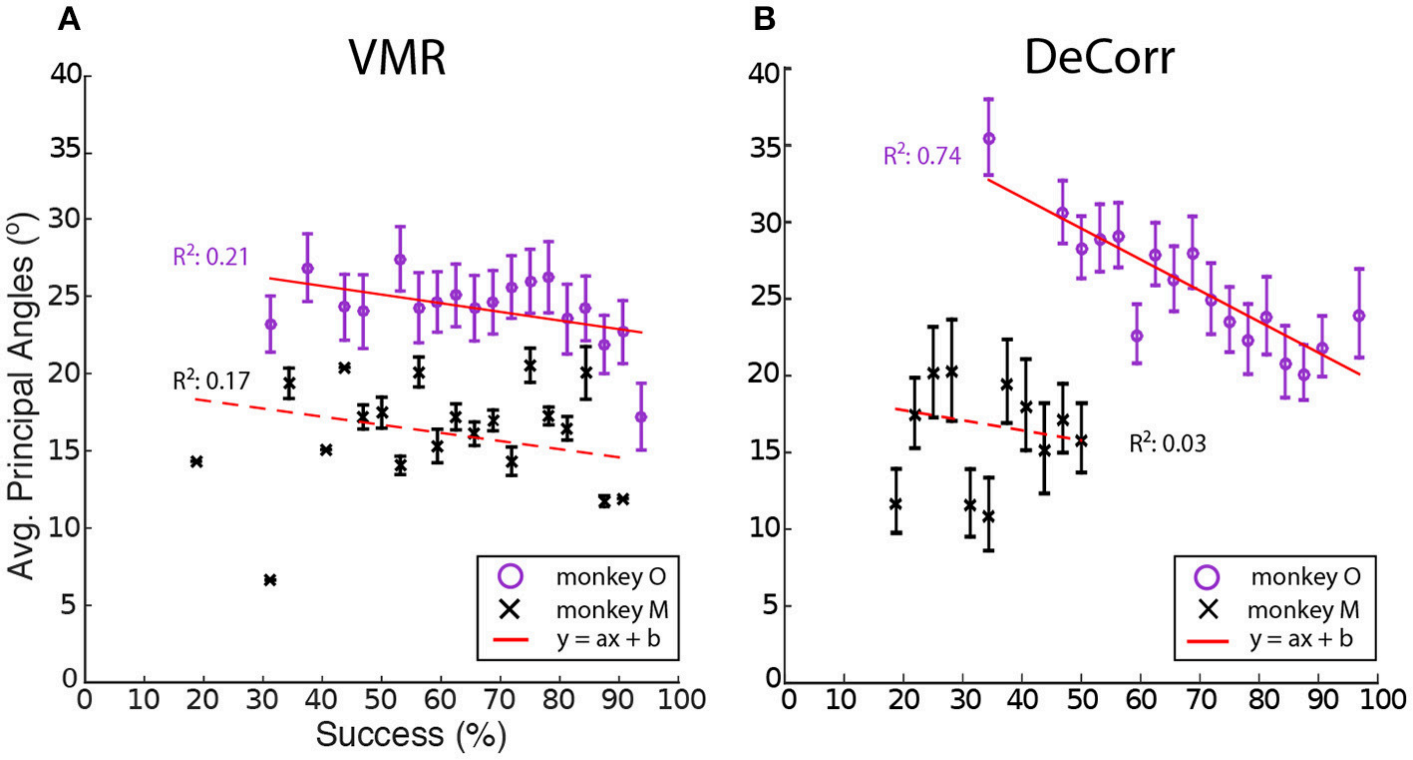
**Average principal angles between baseline and perturbations manifolds**. **(A)** VMR task angles for monkey O (purple circle) and M (black cross) vs. success rate for all sessions. Error bars show standard error across all trials. Red line shows fit to decreasing model (*y* = *ax* + *b*). **(B)** DeCorr task angles for both subject vs. success rate for all sessions. Error bars show standard error across all trials.

We computed the average PAs between the baseline and perturbation (VMR and DeCorr) manifolds, shown in Figure 6. Overall we observed a decreasing trend in these average angles as performance improved, a simple linear model explained some of the variability in the DeCorr trials (*R*^2^ ≥ 0.5) for one subject. A similar trend was measured in VMR trials, although with a poor model fit. However, we consider this decreasing trend captures the overall shifts in PAs, where larger shifts occur when the perturbations are first introduced. These changes in the manifolds could reflect both how the neurons interact with each other, and how inputs to these neurons are varying their control signals throughout the tasks. We would expect that changes due to controller inputs would stay closer to the original manifold, similar to the within-manifold task described by Sadtler et al. (2014), and these could be measured as smaller PAs between the manifolds.

In order to test whether the perturbation type or the improvement in performance had effects in the average PAs, between the manifolds of baseline and each perturbation, we used one-way ANOVA for circular data (Zar, 1996). We tested the levels of task type (VMR and DeCorr), and the performance in each set of trials. While we found that for one subject performance had a significant effect in the average PAs [*F*_(20, 157)_ = 1.73, *p* = 0.033], the perturbation type did not have a significant effect for either of the subjects (*p* ≥ 0.5), and we found no significant interactions between task type and performance level.

## 4. Discussion

It is clear that motor learning and adaptation are captured in changes in cortical mapping of movement (Taylor et al., 2002; Paz and Vaadia, 2004; Wahnoun et al., 2006; Jarosiewicz et al., 2008; Ganguly and Carmena, 2009; Ganguly et al., 2011). Neurons can change basic tuning properties like preferred directions (Paz and Vaadia, 2004; Jarosiewicz et al., 2008), and can even adapt to entirely arbitrary mappings between neural activity and motor output (Ganguly and Carmena, 2009). Here we describe findings which show that although mapping outcomes can be extremely different (e.g., uniform vs. non-uniform shifts in PDs), part of the neural mechanism for achieving those outcomes is similar.

Neural adaptations and correlates are described here during two very distinct motor learning paradigms: a uniform VMR perturbation, which induced expected errors in movement and behavior (Krakauer et al., 2000; Paz and Vaadia, 2004; Jarosiewicz et al., 2008), and a non-uniform DeCorr task, which generated bigger and more random errors in the behavior (Figure 3). Our major findings indicate important similarities in the mechanisms that allow subjects to reach the solutions for each task. The neural signals display a population wide adaptation to both tasks (Figures 4, 5 and Supplementary Figure 5), which implies there is a global response to both perturbations, and eventual solutions are found for the tasks (Figure 5 and Supplementary Figure 5). We also measured transient changes in the tuning properties of the neural signals, and found that success rate had a more significant effect in these dynamic changes than the type of perturbation (Figures 4, 6). This suggests that learning might be encoded in these transient variations in individual tuning, and agrees with recent findings that motor cortical activity modulates with both movement direction and previous trial success or failure (Yuan et al., 2014).

### 4.1. Directional tuning changes during learning

We found significant correlations between task performance and changes in the aPDs of both tasks (Figure 4). When subjects learn a task, or try to solve a novel problem, there is often an increase in global entropy in systems, and an eventual reduction in this uncertainty as learning progresses (Cordier et al., 1994; Zacksenhouse et al., 2007; Schöllhorn et al., 2009; Suminski et al., 2010). As Cordier et al. (1994) shows, it is possible to measure the entropy in exploration paths (e.g., a rock climber's route), by considering the tortuosity of such trajectories. We consider that an analogy could be made with the transient changes in aPDs, and these changes could in part mirror variations in the system entropy, which would suggest an exploratory strategy that the brain engages in when trying to solve challenges posed by a novel task.

Each of our tasks could be solved in principle if the subjects were able to solve the credit assignment problems offered by the two perturbations. In order to verify whether the subjects were able to solve the credit assignment problems, we measured the final changes in aPDs from baseline, as shown in Figure 5 and Supplementary Figure 5. In the VMR task, the preferred directions yielded expected shifts across the entire neuronal ensemble (Figures 5A,C, and Supplementary Figures 5A,C), which would correspond to solving the credit assignment problem. However, this was a global perturbation and could be led by structures that drive motor cortex. Uniform tuning changes across the neural units hint to changes in the controller, rather than in the internal dynamics of these units. To verify this, we also measured possible re-aiming strategies using the latent target calculation developed by Chase et al. (2010), which can estimate new target directions that better explain the firing rate changes in the recorded neurons (See Supplementary Material for a description of the method). Using this algorithm, we found that changes in firing properties during VMR trials were indeed explained by re-aiming strategies, where the new targets were on average shifted 30° in the opposite direction of the VMR (Supplementary Figure 6). For the majority of the VMR sessions (monkey O: 33/41, μ = 39.57°; monkey M: 17/17, μ = 40.54°) the latent directions had shift significant around 30° (circular mean test, Zar, 1996, *p* ≤ 0.05).

In the DeCorr task, such a simple global solution was not possible. The latent target directions did not follow a single direction as in the VMR trials (see Supplementary Figure 6), and the majority of the trials had significantly different target locations between baseline and DeCorr trials (monkey O: 20/21, μ = 41.83°; monkey M: 3/3, μ = 57.06°; circular mean test, Zar, 1996, *p* ≤ 0.05). The only “correct” solution would be to identify those few neurons for which we had altered the cPDs, and change the system to use those neurons with their new assigned preferred directions. Instead, the entire population had significant aPD shifts during the DeCorr task, without the uniformity we observed in the VMR case (Figure 5B and Supplementary Figure 5B). This non-uniformity was expected, since the task itself was designed to disrupt the neuronal dynamics in a non-uniform manner. However, we did not observe adaptation only on specific subsets of neurons, as previous groups have reported (Jarosiewicz et al., 2008), but rather compensations distributed across the entire neuronal ensemble. We observed larger shifts in the aPDs of the rotated neurons during the DeCorr task for one subject(μ_*Rot*_ = 31.71°), when compared to the non-rotated sub-population (μ_*Non*−*rot*_ = 18.65°), as shown in Figure 5D. However, if we measure the shift in the rotated neurons only in direction of the perturbed cPDs, we observe a smaller and non-significant shift. Interestingly, the second subject had larger, albeit not significant (Watson-Williams test, Zar (1996)), shifts in the non-rotated sub-population (μ_*Rot*_ = 39.78°; μ_*Non*−*rot*_ = 53.77°). This subject was not able to fully adapt to the task, so these larger changes in the non-rotated neurons provide a snapshot of the ensemble wide variations during the learning process. In other words, while the perturbed cells did have larger final aPD shifts, they were not always in the directions required to compensate for the DeCorr perturbation as would be expected if the brain was solving the credit assignment problem (Paz and Vaadia, 2004; Jarosiewicz et al., 2008), and were most likely the result of initial global shifts across all the neural population. Finally, although the DeCorr perturbation improved the uniformity of the cPD distribution (see Supplementary Figure 1), this did not translate to an immediate improvement in performance, as seen in the initial drop in performance for the two subjects after the perturbation was introduced (Figure 3).

### 4.2. Changes in population dynamics correlate to task improvement

We found that cross-correlation coefficients did vary between baseline and each task, but these changes were not consistent across the different sessions and subjects, and we did not measure any significant differences between the rotated and non-rotated pairs in the behavior of their cross correlations. These results suggest that the changes in neural dynamics were more dependent on the stage of the learning process than on the type of task the neural system was trying to solve.

In addition to changes in preferred directions or cross correlations, it is possible that wider changes could be observed in the underlying space of the neural representations of the tasks. It is conceivable that a change in the neural space would not be directly reflected in changes in aPDs, and so we estimated reduced neural spaces for the tasks and tasks states. In order to further characterize changes in neural dynamics during learning of both tasks, we measured the principal angles between these subspaces during baseline and each perturbation. In Figure 6 we show transient changes in average PAs as subjects learned the perturbation tasks (VMR and DeCorr), observing an inverse relationship between PAs and performance during both tasks. While we expected to find very distinct ranges in the PAs of the two tasks, we were surprised to find similar ranges and relationship between PAs and performance. However, in the DeCorr perturbation, we found a shift from small to large PAs (0–30° to 60–90°, data not shown), indicating that the system could be operating in a separate neural space (Sadtler et al., 2014). Thus, it would appear that the DeCorr task required larger changes in the intrinsic manifold of the neural signals. However, it is hard to interpret changes in the underlying manifolds from average activity, and further studies at single trial level would help us track these changes. We assume that if more neuron pairs were rotated, we might be able to measure larger changes in the manifolds of baseline and perturbed tasks, although the subjects would possibly need more time to bring performance back to baseline.

As Sadtler et al. and Ranganathan et al. have already shown, there might be some internal constraints in the neuronal ensemble that make it more or less likely for a subject to become proficient at a task. Our results suggest that indeed there are certain limitations as to how much can we ask the neuronal population to change its internal mapping or dynamics. Keeping a stable map across several days allows subjects to adapt and learn new and complicated mappings between neuronal activity and desired output. However, this certainly does not seem to be enough to alter underlying functional relationships across different neural units, at least not for the long term as is shown in our results (Figures 4, 6).

The similarities between the two tasks and the analogous neural changes during learning give evidence that the neural circuitry engaged similar strategies when adapting to each task, even though the “correct” solution is quite different between the tasks. In other words, a similar random exploration in the neural space could be driving the adaptation in both learning challenges, and the harder the solution might be, or the more local minima there might be in the neural space, the longer it would take for the subjects to find a solution. Our results also suggest that underlying functional connections between neural units are not easily decoupled, so it is understandable that a task that requires this from the system will take longer to learn.

Overall, our results show that the brain uses similar strategies to solve strikingly different tasks. We compared how the neural signals changed as the monkeys adapt to each task, and showed the transient and final changes in preferred directions (Figures 4, 5 and Supplementary Figure 5). Moreover, we believe that the similar global changes in cross-correlation coefficients also hint that similar strategies are used when adapting to the two tasks (Supplementary Figure 7 and Supplementary Table 2). It remains unclear whether these similarities in the adaptation process might interfere with learning, or if subjects will still be able to perform the tasks when alternating between perturbations within a single session, as suggested by Ganguly and Carmena (2009). Similarly, our results indicate that system wide changes are responsible for task adaptation, so these processes should be measured in tasks that allow the exploration of neural ensembles as a modular system. Experiments which explore these system wide variations can provide better information about dynamic adaptations in neural systems and reveal limitations to the challenges the brain can solve.

## Author contributions

All authors listed, have made substantial, direct and intellectual contribution to the work, and approved it for publication.

## Funding

Grant support R01 NS063372 01.

### Conflict of interest statement

The authors declare that the research was conducted in the absence of any commercial or financial relationships that could be construed as a potential conflict of interest.
