# Supplementary material for: Uniform and Non-uniform Perturbations in Brain-Machine Interface Task Elicit Similar Neural Strategies
Source: Front Syst Neurosci. 2016 Aug 23;10:70. doi: 10.3389/fnsys.2016.00070 (PMC4994425; doi:10.3389/fnsys.2016.00070)
Supplement: Supplementary file 1 [file DataSheet1.pdf]

Supplementary Material: Uniform and Non-uniform Perturbations in Brain-Machine Interface Task Elicit Similar Neural Strategies

Michelle Armenta Salas, Stephen Helms Tillery*
*Correspondence:
Stephen Helms Tillery:
steve.helmstillery@asu.edu

1 SUPPPLEMENTARY FIGURES

1.1 Decorrelation perturbation

Supplementary Fig. 1 shows examples of changes in preferred direction (PD) distribution, measured as the angle differences between the neurons, before (black bars) and after (purple bars) the decorrelation (DeCorr) perturbation. We produced significant shifts in the angles of the rotated pairs, even though only one neuron of each pair was rotated (Fig. 1B). However, we did not observe any significant changes in the overall angles of all the cell pairs (Fig. 1A). The average shift between the baseline and the perturbed PDs for all the neurons were between 4-15°.

1.2 Control for chance performance

Supplementary Fig. 2 shows examples of the effects of active assistance in the task for both subjects, where the purple trace shows the average distance to target across the simulated trials (shaded area represents standard error). A minimum distance of seven centimeters was considered a target hit. The effects of the active assistance were larger for monkey O (left panel) than for monkey M (right panel); however, they were not significant enough to explain the success rate of the monkey. Bottom right insets display the cumulative distribution functions of angles between the target and cursor, for the simulated (red) and the actual (purple) population vectors (p¡ 0.001, one-sided two-sample K-S test).

1.3 Changes in tuning preferences after perturbations

Supplementary Fig. 3 displays weighted PDs contribution (gray arrows) to the population vector, in order to produce movement (red arrow) towards a specific target (dark circle). With each cursor movement, we can update the direction to the desired target (black arrow) to re-estimate possible changes in the neurons PDs. Figure 3A shows arbitrary PDs with the corresponding movement vector, and target direction. It is possible for neurons to take a number of strategies after a perturbation is introduced to the system. Some of the proposed strategies are re-weighting of the neurons contribution to the movement, re-aiming to a latent/virtual target, or re-mapping (Jarosiewicz et al., 2008). We expect to measure a mix of these strategies in our neurons, however in the visuomotor rotation (VMR) trials we consider that the compensation would be lead by re-aiming strategies. Figure 3B shows what we would observe if the PDs did not show any adaptation after the rotation: the cursor movement would shift in the direction of the rotation, and the

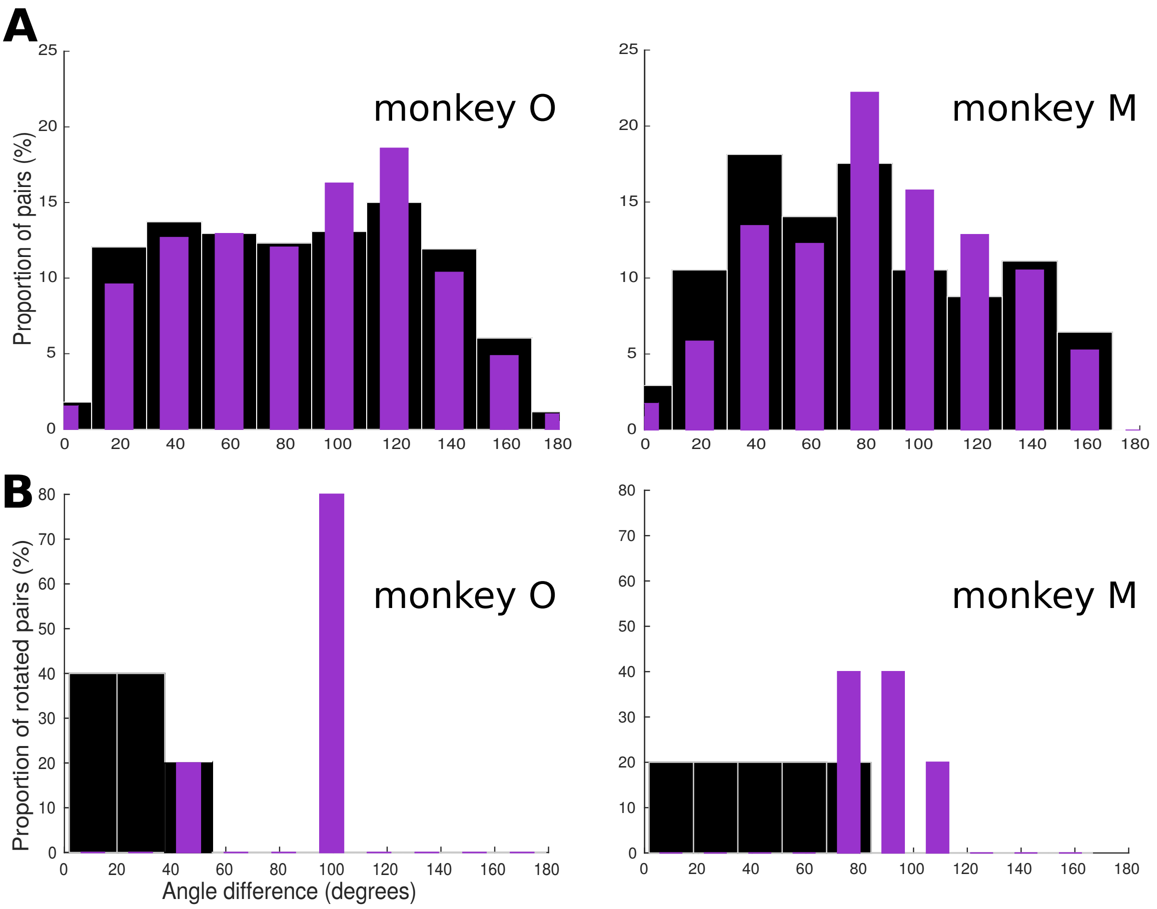

Supplementary Figure 1. Changes in preferred directions distributions in DeCorr perturbation. **A:** Distribution of angle differences between neurons used for active brain control for a single baseline session. Histograms display the percentage of cell pairs vs. angle in degrees. **B:** Distribution of angle differences between cell pairs before (black) and after (purple) the DeCorr perturbation.

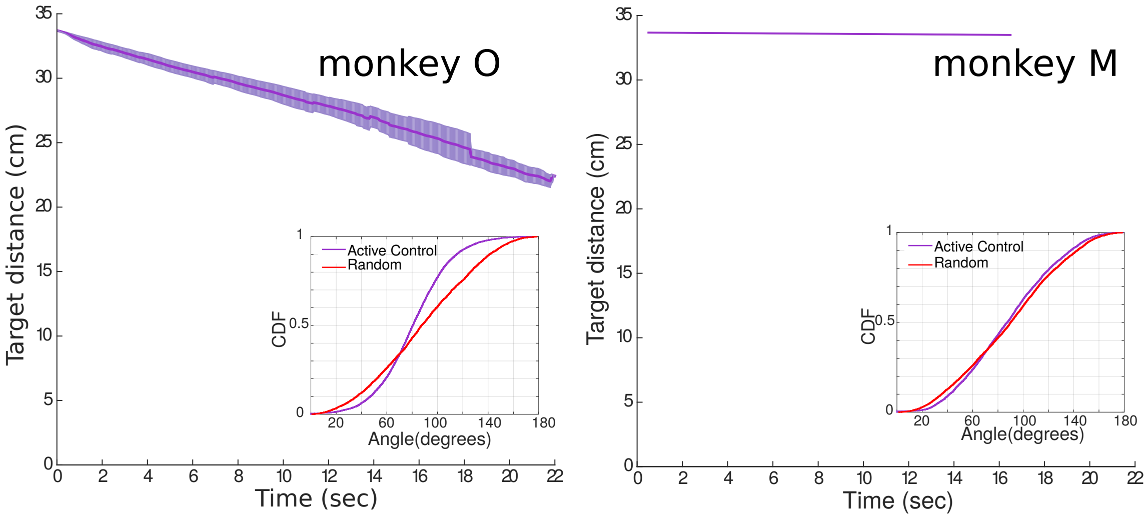

Supplementary Figure 2. Control for chance performance. Simulated movements from shuffled cPDs from monkey O (left panel) and monkey M (data). Average distance to target if maximum trial time was allowed. Shaded areas represent mean +/- one standard deviation. Bottom right inset in each panel displays the CDF of angles between PV and target from the active brain control (purple trace), and the simulated movements (red trace).

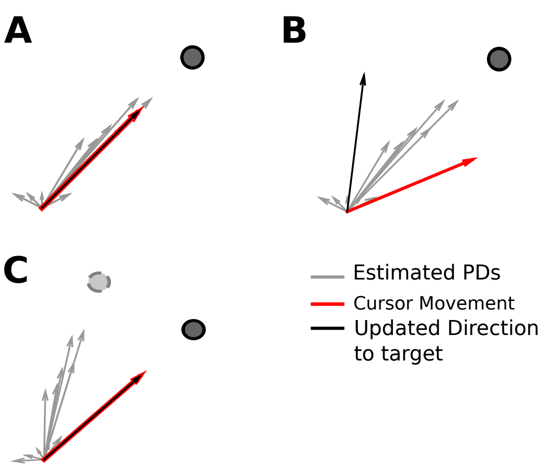

Supplementary Figure 3. Possible adaptations in aPDs during BMI-control. **A**: Unperturbed case, weighted aPDs (gray arrows) produce movement displayed in unitary red vector towards the target (dark circle). Target direction from cursor position (black arrow) matches the actual target direction. **B**: VMR case without modulation, unchanged aPDs (gray arrows) produce rotated movement vector (red arrow), and shifted target direction (black arrow). **C**: VMR case with modulation, aPDs re-aim to virtual target (dashed gray circle), producing red movement cursor, and direction to target that matches the actual target position.

Supplementary Table 1. Summary of sessions with fixed/new decoders for each task in both subjects.

Task type	Monkey O	Monkey M
Decoder updates (weeks)	1, 3, 5, 10, 12, 13, 15(2), 16, 17, 19(2).	1, 2, 3, 4, 5
VMR (sessions)	Total: 41 New map: 8 Fixed map: 33	Total: 17 New map: 4 Fixed. map: 13
DeCorr (sessions)	Total: 21 New map: 4 Fixed map: 17	Total: 3 New map: 1 Fixed map: 2

updated direction to target would shift in the direction opposite to the rotation. Figure 3C shows changes in the PDs under the re-aiming strategies, where the neurons aim towards a virtual target (dashed light circle) which produced a cursor movement in the direction of actual the target, and a matching direction to target.

Supplementary Table 1 summarizes the total number of sessions ran for each monkey, the number of sessions where a new calibration map was used ("New map"), and the weeks from the beginning of the experiment when these updates occurred, and the number of sessions where the calibration map from the previous session was utilized ("Fixed map"). For each subject, the beginning of the experiments marks "week 1", parenthesis numbers indicate two or more decoder updates in that week (e.g. week 15(2), means two decoder calibrations in week 15).

1.3.1 Preferred directions in baseline trials

We expected the neural units to change their tuning throughout the sessions and across several days, but considered that, similar to the behavior, these neural tunings would stabilize during the baseline unperturbed trials across days. Taking the action preferred directions (aPDs), estimated during active brain control, we

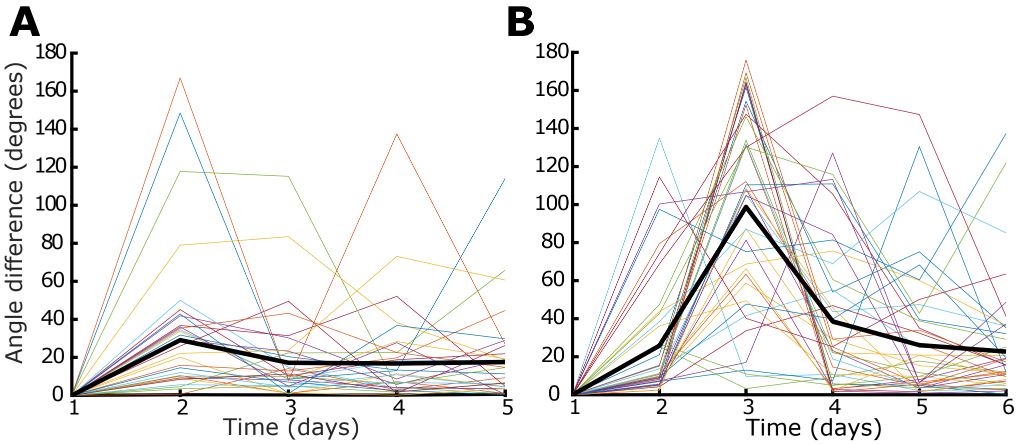

Supplementary Figure 4. Shifts in baseline preferred directions. **A**: Angular shifts in aPDs during VMR across five sessions. Each line represents a different neural unit, the black thick line shows the average shift across all units. **B**: Angular shifts during DeCorr trials, neural units displayed are different than those in panel A. Plot has the same format as panel A

tracked the changes of these baseline aPDs across different sessions with the same decoder. Supplementary Fig. 4 shows example angular shifts in preferred directions across days with stable decoders for monkey O, during VMR and DeCorr tasks. The angles were measured between the aPDs of consecutive days.

Most sessions had larger shifts between the first and second days, in both subjects, and the angle shifts followed a decreasing power curve trend ($R^2 \geq 0.8$) for most of these sessions (monkey O: 38/62; monkey M: 13/20).

1.3.2 Preferred direction shifts during learning challenges

Supplementary Fig. 5 displays exemplary data similar of final shifts in aPDs for monkey M. Figures 5A and 5C show aPDs shift after VMR adaptation, where the majority of neurons had shifts that followed the applied perturbation. Figures 4B and 5D show similar trajectories and pooled data during the DeCorr trials, respectively. Rotated neurons are highlighted (magenta traces/bar) from non-rotated pairs (blue/black).

1.3.3 Latent target estimation

In order to estimate possible re-aiming strategies used in either task, we implemented the algorithm proposed by Chase et al. (2010), where a new virtual or latent target direction is estimated, such that it would better explain the changes in the firing activity of the neural units (Supplementary Fig. 3 C). The method assumes that the neural units firing activity follows a cosine tuning function, it calculates a new set of preferred directions for each neural unit based on the average trajectories for each target direction during online active brain control. After these initial movement-based tunings were calculated, an iterative process was used to estimate the latent target positions which most likely described the firing rates. The estimated latent target direction $\vec{d}_j^*$ was computed such that the error between the firing rates measured for the *jth* target and the firing activity predicted by the new tuning curves was minimized, as stated in 1(Chase et al., 2010).

$$\vec{d}_j^* = \arg\min_{\vec{d}} \left(\sum_{i=1}^{N} \frac{(y_{i,j} - \lambda_i(\vec{d}))^2}{\sigma_i^2} \right), \tag{1}$$

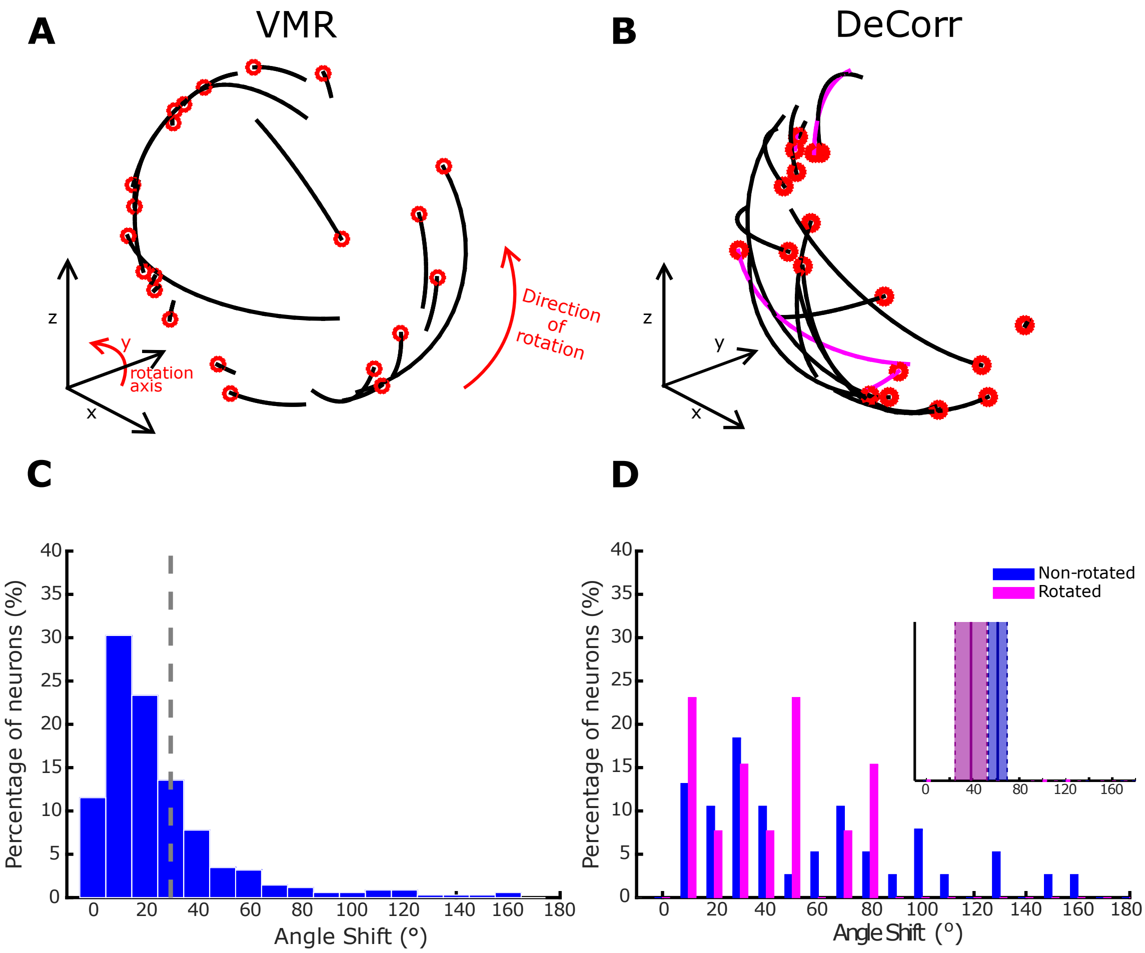

Supplementary Figure 5. Preferred Direction Changes in VMR and DeCorr task. **A:** aPDs changes during VMR task (CCW direction): aPDs during baseline (black end) and after perturbation (red dot) are displayed for each neuron used for brain control. **B:** aPDs changes during DeCorr task (4 rotated pairs): aPDs during baseline (black end) and after perturbation (red dot) are displayed for each neuron used in brain control, magenta trajectories highlight the rotated neurons. **C:** Shift in neurons aPDs, pooled data are displayed for all VMR sessions for monkey M. Dashed gray line illustrated average shift. **D:** Shift in neurons aPDs, pooled data for all DeCorr trials for monkey M. Blue bars show data for non-rotated neurons, and magenta bars display data for rotated neurons.

where $y_{i,j}$ refers to the measured firing activity of the *ith* neuron in the direction of the *jth* target, $\lambda_i(\vec{d})$ is the re-estimated movement based tuning curve of the *ith* neuron evaluated at $\vec{d}$, and σ_i^2 is the variance of the *ith* neuron.

After estimating initial latent target directions for each target, we re-estimated the tuning curves using these new directions. Then repeated the latent target and tuning curve estimation steps until the error in the average tuning curves estimation was less than 1%, similar to that used in Chase et al. (2010).

Similar to the preferred direction calculation, we used sets of trials across different days, only using successful trials, we computed these for the baseline, VMR and DeCorr task of both subjects. Supplementary Fig. 6 shows exemplary locations of the new estimated targets during baseline trials and during both perturbation tasks for both subjects.

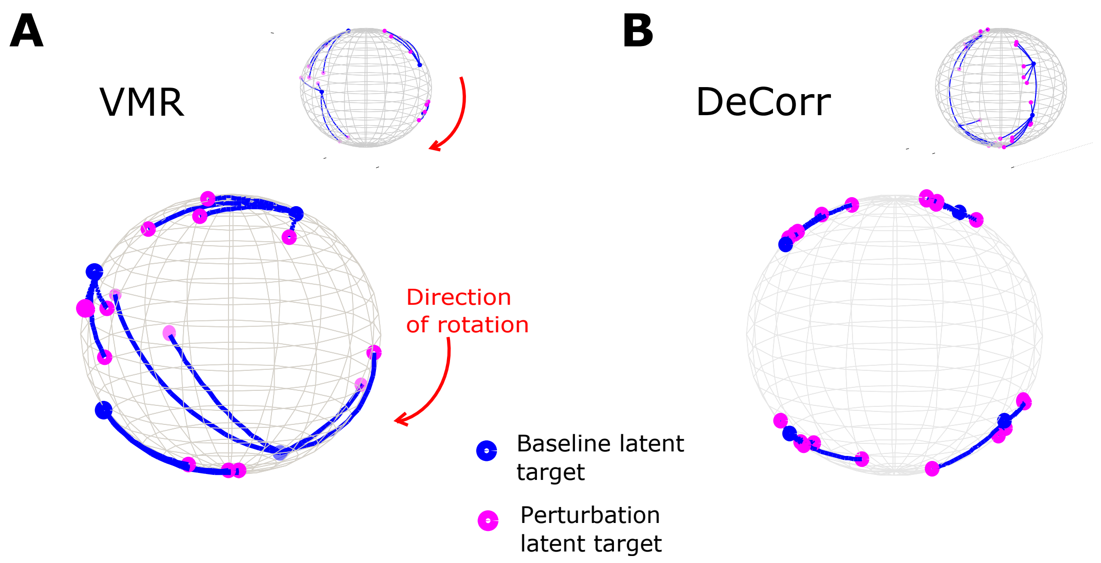

Supplementary Figure 6. Latent target directions. **A:** Estimated latent directions during baseline (blue circles) and VMR trials (pink circles) for subject O and M (top inset). Each pink circle shows the estimated target direction across different blocks of trials. All trials had a clockwise visual rotation. **B:** Estimated latent directions for DeCorr trials, same format as panel A, top right inset shows data for monkey M.

1.4 Changes in neural population dynamics

Supplementary Fig. 7 shows examples of variations in the average peak cross-correlations values during DeCorr trials for both subjects. We see significant shifts after first introducing the perturbation. However, as we track the peak correlations across sessions, we observe eventual convergence to baseline values in both subjects.

In addition, Supplementary Table 2 shows results from one-way analysis of variance test of peak cross-correlations for both subjects. We tested whether task type (baseline vs DeCorr) had an effect in the peak cross-correlation values across all cell pairs. We found the majority of the sessions had significant shifts in these peak values, and higher cross-correlations were measured during baseline trials, when compared to DeCorr trials.

1.4.1 Factor analysis methods

We used factor analysis (FA) algorithm to reduce the dimensions of the neural space, and estimate latent dimensions that explain the variability of the neural firing activity. We based this analysis using the expectation-maximization (EM) algorithm developed by Rubin and Thayer (1982), and applied to neural events by Yu et al. (2009). This algorithm allows to estimate factors or unobservable dimensions from observable measurements. The EM algorithm applied to FA allows to assume that the observable measurements, the firing activity of neurons, will depend in the activity of unobservable dimensions. Their relationship is described in (2).

$$u \mid z \sim N\left(\Lambda z + \mu, \Psi\right), \tag{2}$$

where u represents the firing activity of the recorded neurons, z refers to the latent variable, which follow a Normal distribution with zero mean and unitary variance (see (3)). The firing of the measured neural units is conditioned by the latent variables, following a Normal distribution with mean $\Lambda z + \mu$, where Λ

Supplementary Table 2. Statistical test for peak cross-correlation changes. One-way ANOVA results for both subjects across all DeCorr sessions

Subject	Trials (units rotated)	Test results	Average comparison
Monkey O	224 (6 units)	$F_{1,990}$ = 34.41, p < 0.001	baseline > DeCorr
	160 (6 units)	$F_{1,990}$ = 107.31, p < 0.001	baseline > DeCorr
	192 (6 units)	$F_{1,990}$ = 137.45, p < 0.001	baseline > DeCorr
	224 (6 units)	$F_{1,990}$ = 122.44, p < 0.001	baseline > DeCorr
	192 (6 units)	$F_{1,990}$ = 114.74, p < 0.001	baseline > DeCorr
	224 (6 units)	$F_{1,990}$ = 78.93, p< 0.001	baseline > DeCorr
	256 (6 units)	$F_{1,990}$ = 114.74, p < 0.001	baseline > DeCorr
	256 (5 units)	$F_{1,1558}$ = 63.98, p < 0.001	baseline > DeCorr
	224 (5 units)	$F_{1,1558}$ = 38.99, p < 0.001	baseline > DeCorr
	256 (5 units)	$F_{1,1558}$ = 149.20, p < 0.001	baseline > DeCorr
	256 (5 units)	$F_{1,1558}$ = 84.03, p < 0.001	baseline > DeCorr
	224 (5 units)	$F_{1,1558}$ = 43.71, p < 0.001	baseline > DeCorr
	288 (5 units)	$F_{1,1558}$ = 0.24, p = 0.620	N/A
	256 (5 units)	$F_{1,550}$ = 3.76, p = 0.053	N/A
	224 (5 units)	$F_{1,550}$ = 15.14, p < 0.001	DeCorr > baseline
	224 (5 units)	$F_{1,550}$ = 2.51, p = 0.114	N/A
	256 (5 units)	$F_{1,550}$ = 1.08, p = 0.300	N/A
	192 (5 units)	$F_{1,754}$ = 15.75, p < 0.001	DeCorr > baseline
	224 (5 units)	$F_{1,700}$ = 19.52, p < 0.001	DeCorr > baseline
	192 (5 units)	$F_{1,754}$ = 62.70, p < 0.001	DeCorr > baseline
	256 (5 units)	$F_{1,753}$ = 40.14, p < 0.001	DeCorr > baseline
Monkey M	288 (5 units)	$F_{1,299}$ = 9.37, p = 0.002	DeCorr > baseline
	288 (5 units)	$F_{1,318}$ = 8.79, p = 0.003	DeCorr > baseline
	288 (5 units)	$F_{1,319}$ = 0.020, p = 0.887	N/A

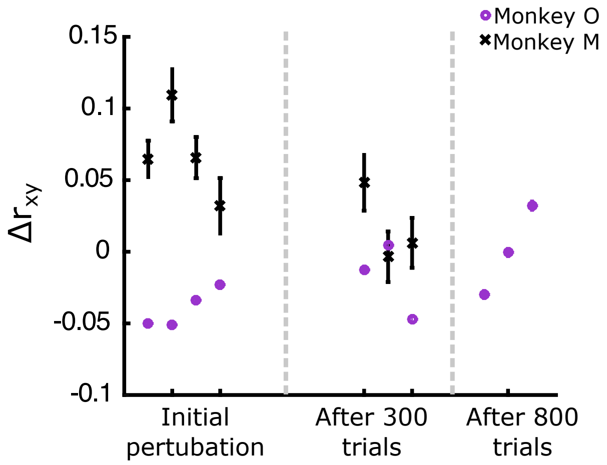

Supplementary Figure 7. Changes in peak cross-correlations. Average shift from baseline for monkey O (purple circle) and monkey M (black cross) across multiple sessions. Initial perturbation shows the first 4 blocks (128 trials) after DeCorr perturbation was introduced. Then 300-400 trials after perturbation in the same set of neurons, and after 800 trials.

contains the weights that relate the latent dimensions with the neural units in its column space, and Ψ is the covariance, as shown in (2):

$$z \sim N(0, I)\,. \qquad (3)$$

The EM algorithm allows to estimate the Λ, which contains the manifold that relates the latent variables with the neural activity in its column space. The subspace $\Lambda \in \mathbb{R}^{p \times q}$, where p is the number of recorded neurons, and q the number of latent dimensions, can help describe neural activity and task parameters.

This EM algorithm has been implemented for FA by Ghahramani (1996) in MATLAB (Mathworks, Inc.), and more recently applied to neural process by Yu et al. and Cowley et al. (2013). We formatted our neural data as specified in the tutorials for the DataHigh visualization tool, and extracted the estimated intrinsic manifolds from sets of 32 trials. We further use the cross-validation option in the algorithm to estimate the number of dimensions which would maximize the log-likelihood (LL) function of the EM algorithm, and selected the dimensions where, across different days, the LL value had plateaued. Supplementary Fig. 8 shows these LL values across different latent dimensions for the initial set of days. The blue dashed lines highlights the selected dimensions (n = 12).

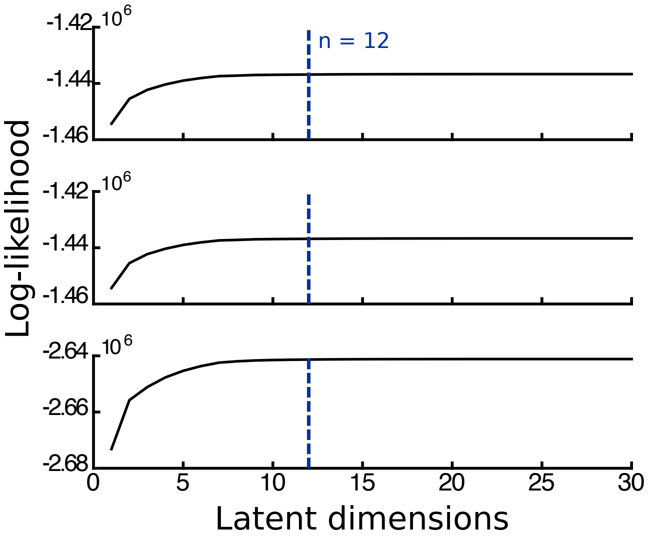

Supplementary Figure 8. Latent dimension selection. Log-likehood (LL) values during expectation-maximization iterations across 12 sessions for one subject, each subplot has 3-4 different sessions. Dashed blue lines highlight the value of the LL when latent dimensions equal 12.

REFERENCES

Chase, S. M., Schwartz, A. B., and Kass, R. E. (2010). Latent Inputs Improve Estimates of Neural Encoding in Motor Cortex. *J of Neuroscience* 30, 13873–13882. doi:10.1523/JNEUROSCI.2325-10.2010

Cowley, B. R., Kaufman, M. T., Butler, Z. S., Churchland, M. M., Ryu, S. I., Shenoy, K. V., et al. (2013). DataHigh: graphical user interface for visualizing and interacting with high-dimensional neural activity. *Journal of Neural Engineering* 10. doi:10.1088/1741-2560/10/6/066012

Ghahramani, Z. (1996). The EM Algorithm for Mixtures of Factor Analyzers

Jarosiewicz, B., Chase, S. M., Fraser, G. W., Velliste, M., Kass, R. E., and Schwartz, A. B. (2008). Functional network reorganization during learning in a brain-computer interface paradigm. *PNAS* 105, 19486 – 19491. doi:10.1073/pnas.0808113105. 00109

Rubin, D. B. and Thayer, D. T. (1982). EM Algorithms for ML Factor Analysis. *Psychometrika* 47, 69–76

Yu, B., Cunningham, J., Santhanam, G., Ryu, S., Shenoy, K., and Sahani, M. (2009). Gaussian-Process Factor Analysis for Low-Dimensional Single-Trial Analysis of Neural Population Activity. *J of Neurophysiology* 102, 2008–2008. 00000
